# ACE2 Is Augmented in Dystrophic Skeletal Muscle and Plays a Role in Decreasing Associated Fibrosis

**DOI:** 10.1371/journal.pone.0093449

**Published:** 2014-04-02

**Authors:** Cecilia Riquelme, María José Acuña, Javiera Torrejón, Daniela Rebolledo, Daniel Cabrera, Robson A. Santos, Enrique Brandan

**Affiliations:** 1 Center for Aging and Regeneration, CARE Chile UC and Department Cell and Molecular Biology, Faculty of Biological Sciences, Catholic University of Chile, Santiago, Chile; 2 Department of Physiology and Biophysics, Biological Sciences Institute, INCT Nanobio-far, Federal University of Minas Gerais, Belo Horizonte, MG, Brazil; Stem Cell Research Institute, Belgium

## Abstract

Duchenne muscular dystrophy (DMD) is the most common inherited neuromuscular disease and is characterized by absence of the cytoskeletal protein dystrophin, muscle wasting, and fibrosis. We previously demonstrated that systemic infusion or oral administration of angiotensin-(1-7) (Ang-(1-7)), a peptide with opposing effects to angiotensin II, normalized skeletal muscle architecture, decreased local fibrosis, and improved muscle function in *mdx* mice, a dystrophic model for DMD. In this study, we investigated the presence, activity, and localization of ACE2, the enzyme responsible for Ang-(1-7) production, in wild type (wt) and *mdx* skeletal muscle and in a model of induced chronic damage in wt mice. All dystrophic muscles studied showed higher ACE2 activity than wt muscle. Immunolocalization studies indicated that ACE2 was localized mainly at the sarcolemma and, to a lesser extent, associated with interstitial cells. Similar results were observed in the model of chronic damage in the tibialis anterior (TA) muscle. Furthermore, we evaluated the effect of ACE2 overexpression in *mdx* TA muscle using an adenovirus containing human ACE2 sequence and showed that expression of ACE2 reduced the fibrosis associated with TA dystrophic muscles. Moreover, we observed fewer inflammatory cells infiltrating the *mdx* muscle. Finally, *mdx* gastrocnemius muscles from mice infused with Ang-(1-7), which decreases fibrosis, contain less ACE2 associated with the muscle. This is the first evidence supporting ACE2 as an important therapeutic target to improve the dystrophic skeletal muscle phenotype.

## Introduction

Duchenne muscular dystrophy (DMD) is a genetic disorder characterized by absence of the cytoskeletal protein dystrophin. This is recapitulated in the DMD mouse model (*mdx*). Absence of dystrophin leads to loss of anchoring of the myofiber to the basal lamina, eliciting subsequent myofiber degeneration that in turn results in progressive loss of muscle mass, weakness, and increased extracellular matrix (ECM) accumulation [1], [2]. Thus, children with this pathology gradually and progressively lose muscle strength, typically requiring the use of a wheelchair from the age of 10 and dying in the late second or early third decade of life as a result of cardiorespiratory arrest. Pathologic features of DMD include myofiber atrophy, fatty accumulation, degeneration, necrosis, and fibrosis, which dramatically affect the environment of the fibers and normal muscle physiology [3]–[6].

Fibrosis is a complex and incompletely understood process characterized by excessive accumulation of collagens and other ECM components [7], [8]. It occurs under chronic disease conditions and affects various tissues and organs [9]. Several findings support the notion that fibrosis directly contributes to progressive muscle dysfunction and the lethal phenotype of DMD [10]–[13].

Targeting the renin-angiotensin system (RAS) is one of the most common therapeutic approaches in cardiovascular medicine [14]. Traditional pharmacologic RAS intervention seeks to prevent excessive AT1 receptor stimulation by repressing synthesis of angiotensin II (Ang II) through inhibition of renin or angiotensin-converting enzyme (ACE) or by direct blockade of the AT1 receptor. This strategy has been successful in cardiovascular therapy, as documented by numerous clinical studies demonstrating reduced mortality and morbidity in treated patients [15]. In recent years other receptors of the RAS have been better characterized in terms of signaling and physiologic function, revealing the existence of a so-called “alternative” or “protective” RAS including AT2 and Mas receptor [16], [17]. These receptors mediate tissue protective activities that counteract not only the AT1 receptor, but also inflammatory cytokines, growth factors, and proapoptotic stimuli. Stimulation of AT2 receptor or Mas has been shown to ameliorate the course of cardiovascular, renal, immunological, and neurological diseases in multiple experimental models [18], [19]. Thus, AT2 receptors and Mas are now regarded as potential drug targets, and respective agonists are in various phases of drug development [20].

Angiotensin-1-7 (Ang-(1-7)) is an endogenous bioactive peptide metabolite in the alternative or protective arm of RAS. It is primarily derived from angiotensin II processing and has important biological effects including vasodilation [21], [22], inhibition of cell proliferation [23]–[25], antihypertensive [26] and antiarrhythmic [27] activity. The biological actions mediated by Ang-(1-7) are transduced by the Mas receptor [13], [28]. Through pharmacologic and genetic evidence, we have determined that the positive effects of Ang-(1-7) observed in dystrophic mice are associated with the reduction of TGF-β-Smad-dependent signaling through the Mas receptor. We found that infusion or oral administration of Ang-(1-7) in *mdx* mice normalized skeletal muscle architecture, decreased local fibrosis, and improved muscle function *in vitro* and *in vivo*. Consistent with these findings, *mdx* mice infused with Mas antagonist (A-779) or deficient for the Mas receptor show worse muscle architecture, increased fibrosis, and increased muscle weakness compared with *mdx* dystrophic mice [13].

Ang-(1-7) is mainly produced by the action of the enzyme angiotensin-converting enzyme 2 (ACE2), a pleiotropic monocarboxypeptidase capable of metabolizing a range of peptide substrates, including Ang I, Ang II, des-Arg9-bradykinin, apelin-13, and opioids [29]–[32]. Ang-(1-7), the major enzymatic product of ACE2, has been shown to reduce Ang II-induced cardiac hypertrophy and remodeling and pressure overload-induced heart failure [33], [34]. Because evidence supporting the beneficial effects of Ang-(1-7) is accumulating, ACE2 is beginning to be considered as a critical pharmacological target with enormous therapeutic projections.

In this study we investigated the presence, activity, and localization of ACE2 in wt and *mdx* skeletal muscle and in a model of fibrosis induced by chronic damage. All the dystrophic muscles studied showed higher ACE2 activity than wt muscle, and ACE2 was localized mainly at the sarcolemma and to a lesser extent in interstitial cells. Furthermore, we evaluated the effect of ACE2 overexpression in *mdx* muscle and demonstrated a reduction in fibrosis, one of the features of dystrophic muscles together with fewer inflammatory cells infiltrating the tissue.

## Methods

### Ethics Statement

All mouse protocols were conducted in strict accordance and with formal approval of the Animal Ethics Committee of the P. Universidad Católica de Chile.

### Mice and Tissue Collection

C57 wt and *mdx* (C57BL/10ScSn) [35] male mice were used. For tissue sampling, animals were anesthetized and sacrificed by cervical dislocation and the gastrocnemius (GAST), tibialis anterior (TA), and diaphragm (DIA) were dissected and removed. Tissues for western blot analysis and ACE2 activity assays were rapidly frozen in liquid nitrogen. Tissue samples for cryosectioning were frozen in isopentane, cooled in liquid nitrogen, and stored at −80°C until processing [36], [37].

### ACE2 Activity Assay

The ACE2 activity assay was performed as previously described with minor modifications [38]. Tissue was homogenized in ACE2 buffer (75 mM Tris pH 7.5, 0.5 μM ZnCl_2_) in ice with an Ultraturrax (Kinematica, Littau, Switzerland). Tissue extracts were centrifuged at 4°C for 10 minutes at 10,000 g and supernatant was retained. The fluorescence emitted by breakdown of the fluorogenic peptide Mca-Y-V-A-D-A-P-K(Dnp)-OH (10 uM; R&D Systems, USA) was measured in a reaction mix containing 50 ug of total protein from tissue extracts, 0.1 M NaCl, and 10 μM captopril in ACE2 buffer to a final volume of 100 μl. Fluorescence emitted was measured at 2-minute intervals over 46 minutes at 37°C at 320 nM excitation and 405 nM emission (Biotek instruments, Sinergy HT, USA). The slope of the linear range of data represents ACE2 activity (fluorescence units/min) and was corrected for total micrograms of protein. The activity of 0.5 nM recombinant mouse ACE2 (R&D Systems, USA) was used as the positive control. Data were expressed as percentage of *mdx* ACE2 activity relative to wt DIA muscle or as ACE2 specific activity.

### Recombinant Adenovirus Preparation and Large-scale Purification

We used an adenovirus encoding human ACE2 driven by the CMV promoter (Ad5CMVhACE2 IRES GFP; referred to as Ad-hACE2). The control adenovirus encodes GFP driven by the CMV promoter (Ad5CMV GFP; referred to as Ad-GFP). Both were purchased from the Gene Transfer Vector Core, University of Iowa (supported by NIH and the Roy J Carver Foundation, USA). Large-scale production of commercial adenoviruses was performed using infected HEK293 cells [37].

### Cell Culture and Transient Infection

Human embryonic kidney (HEK) 293T cells (ATCC CRL-11268, USA) were maintained in DMEM with 4.5 g/l glucose and supplemented with 10% heat-inactivated fetal bovine serum (HI-FBS), 100 units/ml penicillin, and 100 μg/ml streptomycin. To verify protein expression and enzymatic activity, 10 μl of purified adenovirus in 2 ml DMEM with 2% HI-FBS was added to a 75-cm^2^ flask of confluent 293T cells. After 2 hours, 4 ml of fresh growth media was added and the cells were collected after a further 30 hours. The pellet was resuspended in ACE2 buffer, sonicated, clarified, and the protein concentration was determined for measurement of enzymatic activity or immunoblot analysis.

### Chronic Damage of Skeletal Muscle

TA muscle of 3-month-old wt mice was injected with 50 μl of 0.2% BaCl_2_ once a week for 6 weeks in the same muscle to simulate chronic damage. Muscles were isolated and collected for analysis 2 weeks after the last injection [39].

### Transduction of *mdx* Muscle with Adenoviral Vectors

TA muscles of 12-week-old male *mdx* mice were directly injected with 4×10^11^ viral particles of Ad-hACE2 or Ad-GFP under isofluorane anesthesia. The muscles were dissected and removed at the indicated days post-infection and cryosections were prepared for adjacent serial sections [37].

### Immunoblot Analysis

Muscles were homogenized in 10 volumes of buffer Tris-EDTA pH 7.4 with 1 mM PMSF. One volume of buffer containing 2% glycerol, 4% SDS, and 0.125 M Tris pH 6.8 was added to the homogenates. Aliquots were subjected to SDS gel electrophoresis in 10% polyacrylamide gels, electrophoretically transferred onto PVDF membranes (Millipore, USA), and probed with specific antibodies against ACE2 (ab59351, Abcam, USA), fibronectin (Sigma-Aldrich, USA), tubulin (Sigma-Aldrich), collagen III (Rockland, USA), GFP (Santa Cruz Biotechnology, USA), and GAPDH (Chemicon, USA) [13]. All immunoreactions were visualized by enhanced chemiluminescence (Pierce, USA) [11].

### Immunofluorescence Microscopy

For muscle immunofluorescence, cryosections (7 μm) were fixed in 4% paraformaldehyde, blocked for 1 hour in 4% BSA in PBS, and incubated overnight at 4°C with the following antibodies: anti-ACE2 (sc-20998, Santa Cruz Biotechnology), anti-collagen III (Rockland), anti-collagen I (Abcam,), anti-dystrophin (Sigma), anti-decorin (Thelios, USA), anti-CD68 (Serotec, USA) to stain M1 macrophages, or anti-major basic protein (MBP) to stain eosinophils (Millipore, USA). The corresponding Alexa Fluor 568 or 498-conjugated anti IgGs were used as secondary antibodies. For nuclear staining, sections were incubated with 1 μg/mL Hoechst 33258 in PBS for 10 minutes [37].

### Angiotensin-(1-7) Treatment of Mice

Wt and *mdx* mice were subcutaneously treated with Ang-(1-7) (100 ng/kg*min) using an osmotic minipump (Alzet, Durect Co., 1004 model) for 8 weeks, starting at 12 weeks of age [13].

### Skeletal Muscle Histology and Sirius Red Staining

GAST and TA cryosections were placed on glass slides. Hematoxylin and eosin staining was performed to assess muscle architecture and histology. Total collagen content was detected by staining with 1% Sirius red in picric acid [36], [37].

### Statistical Analysis

The statistical significance of the differences between the means of the experimental groups was evaluated using one-way ANOVA with a post hoc Bonferroni multiple comparison test (Graph Pad Prism 5.00). A difference was considered statistically significant at p<0.05.

## Results

### ACE2 Expression is Increased in Dystrophic Muscle

**Figure 1:**
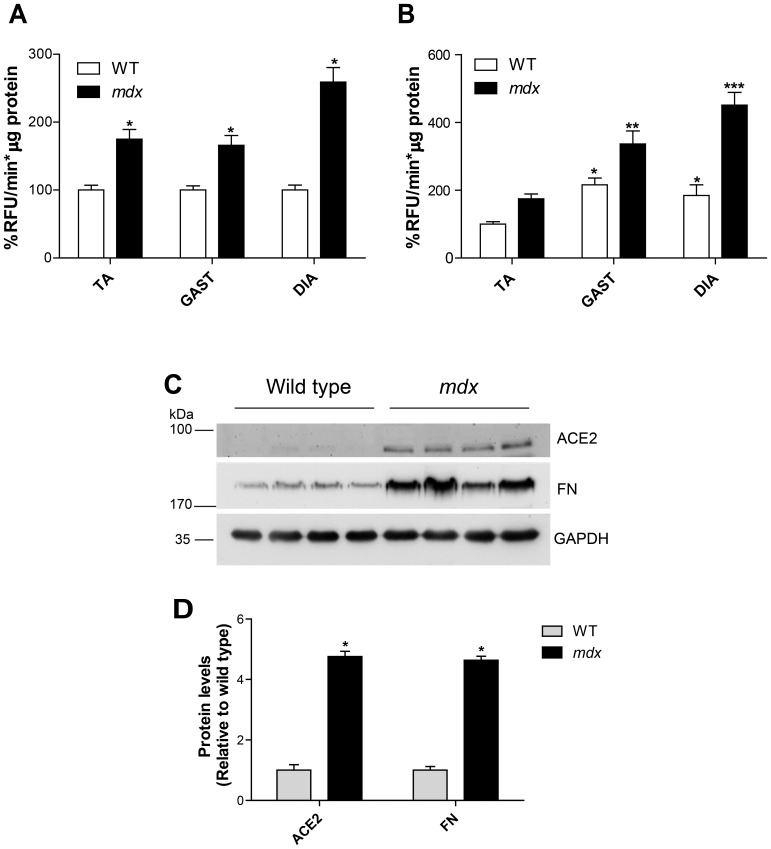
ACE2 activity and protein levels are increased in dystrophic skeletal muscle. (A) ACE2 activity of wt and *mdx* muscle extracts from TA, GAST and DIA. Cleavage rate is expressed as percentage of relative fluorescence units per minute normalized to total protein (%RFU/min*μg protein). Activity of each wt muscle was set as 100%, ANOVA *p<0.05 vs. wt muscle. (B) ACE2 activity of wt TA muscle was set as 100% and compared among all muscles analyzed. Significance between wt GAST and DIA, ANOVA *p<0.05. Significance between *mdx* GAST and *mdx* TA, ANOVA **p<0.05. *mdx* DIA had the greatest activity of all analyzed muscles, ANOVA ***p<0.01. Data are presented as mean ± standard error. TA wt n = 8, *mdx* n = 15; GAST wt n = 7, *mdx* n = 10; DIA wt n = 9, *mdx* n = 13. (C) Wt (lanes 1–4) and *mdx* (lanes 5–8) muscle extracts were analyzed by western blotting to detect ACE2, fibronectin, and GAPDH (loading control). Molecular weight standards are shown on the right. (D) Levels of ACE2 and fibronectin were quantified using relative expression compared with wt; data are mean ± standard error. ANOVA * p<0.001 wt vs. *mdx*.

Protein extracts from wt and *mdx* skeletal muscles were assessed for ACE2 activity by detecting the cleavage of fluorogenic ACE2 substrate. As shown in Figure 1A, ACE2 activity was significantly increased in all *mdx* muscle extracts (black bars) compared to wt muscle extracts (white bars). The greatest increase in ACE2 activity was observed in *mdx* DIA, which had more than 2-fold higher activity than wt DIA. In addition, among the wt muscles studied (TA, GAST, and DIA) the lowest ACE2 specific activity was found in TA extracts (Fig. 1B) and the highest in GAST extracts. These results demonstrate that ACE2 activity is normally present in wt skeletal muscle and is increased in *mdx* skeletal muscles, which might correlate with the onset of the fibrotic response of the dystrophic muscle. Western blot analysis showed increased protein levels of ACE2 and fibronectin, a fibrotic protein that shows increased expression in *mdx* skeletal muscles [13], [37], in *mdx* GAST extracts compared to wt muscle (Fig. 1C). Quantitative analysis showed that ACE2 and fibronectin expression was 4-fold higher in *mdx* muscle (black bars) than in wt muscle (gray bars) (Fig. 1D). Together, the western blot analysis and enzymatic activity clearly indicate that ACE2 expression and activity is significantly increased in skeletal muscle of dystrophic mice.

**Figure 2:**
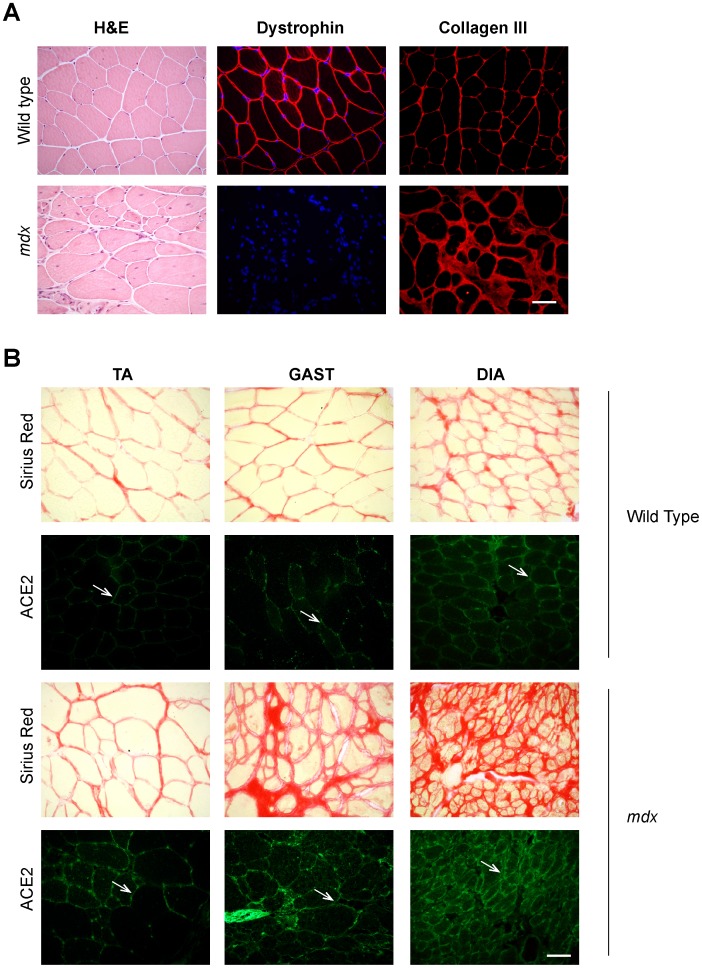
Levels of ACE2 in different skeletal muscle correlate with the degree of fibrosis. (A) Left panels, hematoxylin and eosin (H&E) staining of wt and *mdx* GAST cross-sections; middle panels, immunodetection of dystrophin in GAST; right panels, detection of collagen III by indirect immunofluorescence in cryosections of wt and *mdx* GAST. Nuclei are stained with Hoechst. Bar 50 um. (B) Sirius Red staining of total collagen and indirect immunodetection of ACE2 in cryosections of TA, GAST, and DIA en wt and *mdx* skeletal muscle sections. Arrows indicate ACE2 at the sarcolemma. Bar 50 um.

Next, we evaluated the presence of ACE2 in wt and dystrophic muscles by indirect immunofluorescence. Figure 2A shows the characteristic deterioration of *mdx* muscle highlighted by the presence of fibers with heterogeneous diameter, central nuclei, necrotic areas, and fibrosis as is shown by H&E staining [37]. Figure 2A also shows the absence of dystrophin staining that is characteristic of *mdx* skeletal muscle compared with wt. Fibrosis is revealed by the accumulation of collagen III in *mdx* skeletal muscle compared to wt (Fig. 2A). Figure 2B shows augmented ACE2 staining in all of the muscles studied compared with the corresponding wt muscles. As expected, total collagen determined by sirius red staining was much higher in all of the muscles obtained from *mdx* mouse than in wt muscle. Interestingly, the figure also shows that the less fibrotic muscle (as revealed by Sirius red) correlated with scarce ACE2 detection in the tissue, whereas the more fibrotic muscle correlated with strong detection of ACE2. The results suggest a clear correlation between ACE2 expression and the degree of fibrosis in the muscles analyzed.

### ACE2 Localizes to the Sarcolemma and Interstitial Cells in Fibrotic Skeletal Muscle

**Figure 3:**
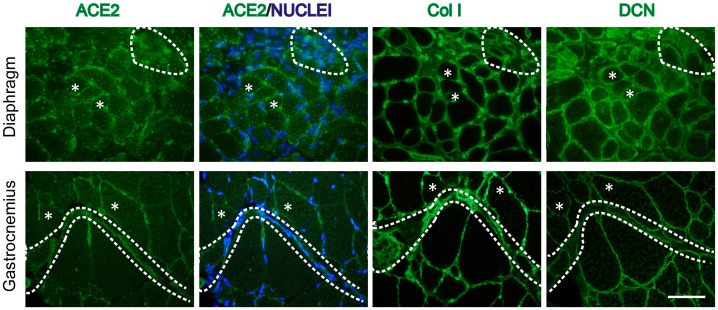
ACE2 localizes at the sarcolemma and interstitial space in dystrophic skeletal muscle. Immunostaining of ACE2, collagen I (ColI), and decorin (DCN) in cryosections obtained from GAST and DIA of *mdx*. Nuclei were stained with Hoechst. The delimitated area shows the same fibrotic tissue zone in three serial sections of GAST and DIA *mdx* muscles, where ACE2 immunostaining is probably associated with interstitials cells. Asterisks show a single fiber, in which ACE2 immunostaining is mainly localized at the sarcolemma. Bar 50 um.

Next we evaluated the localization of ACE2 in skeletal muscle of two animal models in which fibrosis is augmented, the *mdx* mouse and a wt model of induced fibrosis as a result of chronic damage. ACE2 was localized mainly at the sarcolemma of wt muscle fibers (Fig. 2B, arrows) whereas the dystrophic muscle showed augmented ACE2 mainly at the sarcolemma (Fig. 3, ACE2, asterisks) and at the interstitial space, resembling the localization of collagen III described above. ACE2 staining at the sarcolemma was well defined, whereas a more diffuse signal was observed at the interstitial space (Fig. 3, ACE2/Nuclei, delimitated area). The presence of fibrotic material was evident in serial sections stained for the ECM proteins collagen I and decorin (Fig. 3, Col I, DCN, delimitated area). These results suggest that ACE2 in skeletal muscle of dystrophic mice is associated with the skeletal muscle fibers, but is also present in cells within the interstitial space.

**Figure 4:**
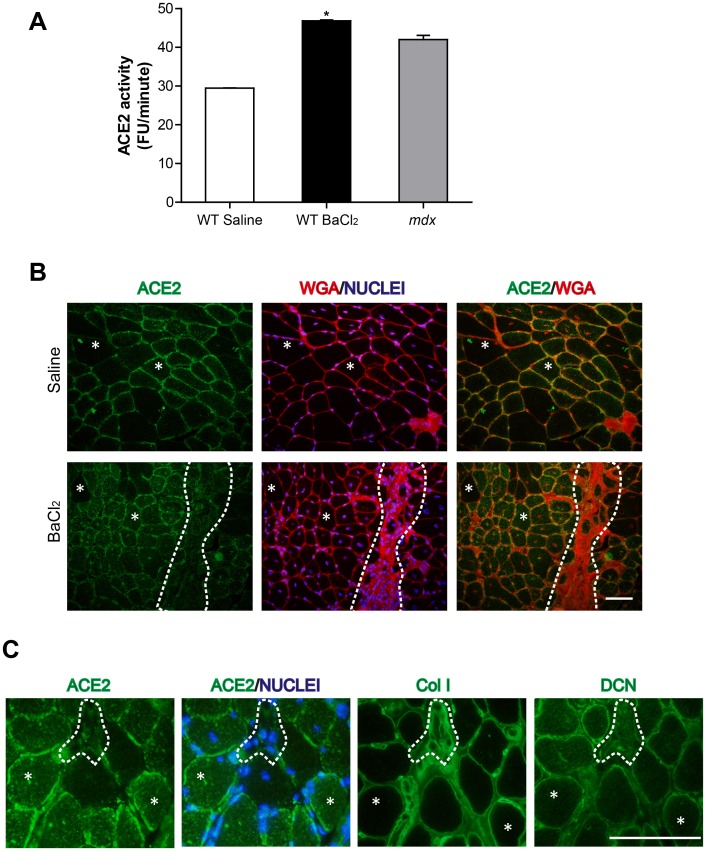
Chronic damage in wt TA increases ACE2 activity. (A) ACE2 activity in extracts from TA injected with saline (WT saline), or with BaCl_2_ (WT BaCl_2_) for 6 weeks, and in *mdx* TA was measured and the cleavage rate expressed as fluorescence units per minute (FU/min). Each sample contained 50 ug of total protein extract. ANOVA *p<0.001 vs. wt saline. (B) Staining of ACE2, WGA, and Hoechst in TA injected with saline (Saline) or BaCl_2_ (BaCl_2_) for 6 weeks. The delimitated area shows a fibrotic tissue zone with relatively low ACE2 immunostaining. Asterisks show a single fiber, in which ACE2 immunostaining is mainly localized at the sarcolemma. Bar 50 um. (C) ACE2, collagen I (ColI), and decorin (DCN) immunostaining in TA injected with BaCl_2_ for 6 weeks. The delimitated area shows a fibrotic tissue zone with interstitial cells, where ACE2 staining is relatively low. Asterisks show a single fiber in which ACE2 is mainly localized at the sarcolemma. Bar 50 um.

To further evaluate whether ACE2 localization on interstitial cells is a general feature of a fibrotic muscle, we examined ACE2 localization in wt TA muscle subjected to induced fibrosis by repeated injections of BaCl_2_ [40]. Six injections at 1-week intervals resulted in significant ECM accumulation 2 weeks after the last injection [39]. Figure 4A shows that ACE2 enzymatic activity increased significantly after the repeated cycles of damage, compared with TA muscle that received saline injections (white versus black bars). The same figure compares ACE2 enzymatic activity in the chronic damage fibrotic model with that in *mdx* TA muscles (gray bars). Interestingly, similar levels of ACE2 activity were observed in both fibrotic models/black vesrsus gray bars). Figure 4B (upper panels) shows immunolocalization of ACE2 in the induced fibrosis model. In TA muscle sections injected with saline, ACE2 was localized mainly at the sarcolemma and co-localized with wheat germ agglutinin (WGA) staining, which labels glycoproteins at the sarcolemma and ECM (Fig. 4B, ACE2/WGA). TA injected with BaCl_2_ (Fig. 4B, lower panels) also showed ACE2 staining mainly at the sarcolemma (asterisks), staining was also present in fibrotic regions, as indicated by the delimitated area. To further examine this, Figure 4C (ACE2) shows a higher magnification image of ACE2 localization at the sarcolemma together with some intracellular staining. The delimitated area shows a zone that contains several nuclei that probably correspond to interstitial cells (Fig. 4C ACE2/Nuclei). Two ECM components, collagen type I (Fig. 4C, Col I) and decorin (Fig. 4C, DCN), were detected in adjacent skeletal muscle sections. Both ECM components are typically found in fibrotic areas and around individual fibers [13], [41]. These results suggest that ACE2 activity increases predominantly at the sarcolemma and probably also in interstitial ECM-producing cells in response to chronic damage (*mdx* and wt fibrotic model).

### Modulation of ACE2 Expression Levels Regulates Fibrosis in Dystrophic Muscle

Given that ACE2 is the major Ang-(1-7) producing enzyme and Ang-(1-7) peptide has anti-fibrotic effects [13], we evaluated whether the fibrosis observed in dystrophic muscle could be reduced by increasing ACE2 activity above the already augmented levels detected in *mdx* skeletal muscle. HEK 293 cells were transduced with an adenovirus carrying the sequence for human ACE2 (Ad-hACE2) or with an adenovirus carrying the sequence for GFP protein (Ad-GFP) as control. Supporting Figure 1 shows an obvious increase in ACE2 activity in protein extracts obtained from cells infected with Ad-hACE2 (black squares), whereas almost no difference was observed in non-infected cells or cells infected with Ad-GFP (gray or white squares). Supporting Figure 1B shows quantitative analyses of the enzymatic assay under the described experimental conditions and Supporting Figure 1C shows protein levels of ACE2, GFP, and tubulin (loading control) by western blot analysis, which was consistent with the increased activity detected in those extracts.

Having demonstrated expression of functional ACE2, we next examined whether it was possible to overexpress the enzyme in TA dystrophic muscle. Supporting Figure 2A shows the kinetics of ACE2 expression in TA infected with adenoviruses carrying ACE2 or GFP several days after injection. Although the enzyme was clearly detectable 1 day after Ad-ACE2 injection, the highest levels were 5 days after. Adenoviral injections have been demonstrated to be inflammatory cues and may explain increased levels of fibronectin five days after injecting Ad-GFP in *mdx* mice. Interestingly, less fibronectin was detected at this time point with Ad-hACE2 injection, suggesting an anti-inflammatory effect of Ang-(1-7) production. Consistent with the western blot data, immunostaining for ACE2 showed low changes in ACE2 levels 1 day after Ad-GFP or Ad-hACE2 injections (Supporting Fig. 2B, top panel). However, 5 days after injection ACE2 staining was stronger at the sarcolemma of individual fibers injected with Ad-hACE2 (bottom panel).

**Figure 5:**
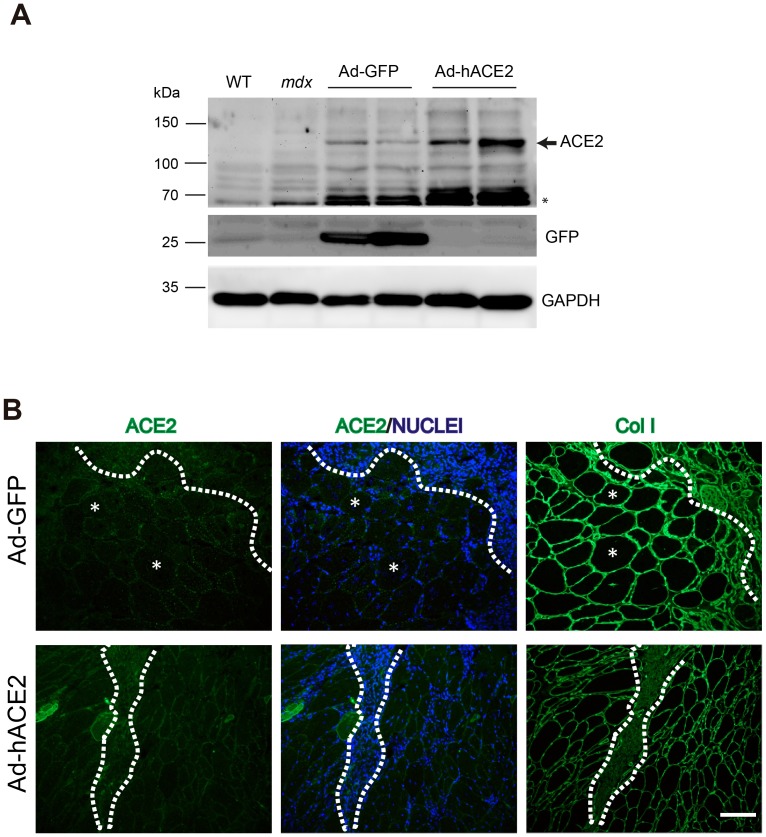
ACE2 overexpression decreases collagen I levels in *mdx* TA muscle. (A) After 5 days of adenoviral injection, half of the TA muscles were analyzed by western blotting to confirm ACE2 and GFP overexpression. Analysis of wt TA, non-injected *mdx* TA, Ad-GFP injected *mdx* TA (n = 2), and Ad-hACE2 injected *mdx* TA (n = 2) is shown. The arrow indicates migration of ACE2 and the asterisk (*) indicates a lower molecular weight band detected by the antibody. (B) The other half of the *mdx* TA muscles from (A) were cryopreserved and analyzed by immunostaining. The upper panel shows serial sections of Ad-GFP injected muscle and the bottom panel sections show Ad-hACE2 injected muscle. ACE2 and collagen I are shown in green and nuclei are shown in blue. The delimitated area marks the injection sites and the asterisks (*) indicate two representative fibers. Bar 50 um.

We next examined whether ACE2 overexpression affected the dystrophic muscle phenotype. Figure 5A shows western blot analysis of ACE2 expression in muscle extracts after 5 days of injection. As expected, a marked increase in ACE2 expression was observed in the muscle extracts injected with Ad-hACE2 (Figure 5A, arrow). A lower molecular weight band was also detected (asterisk), which may correspond to a processed form of the protein. GFP and GAPDH levels (loading control) are also shown. We then investigated whether the expression of collagen in the muscle sections is altered after adenoviral injection. Figure 5B (bottom panel) shows strong staining for ACE2 in the dystrophic muscle injected with Ad-hACE2 and, consistent with the data shown in Supporting Figure 2B, decreased collagen I levels compared with muscle injected with Ad-GFP (Figure 5B, upper panel). These results suggest that the fibrotic phenotype observed in dystrophic muscle could be improved by overexpressing ACE2 beyond the augmented levels detected in the *mdx* muscle.

**Figure 6:**
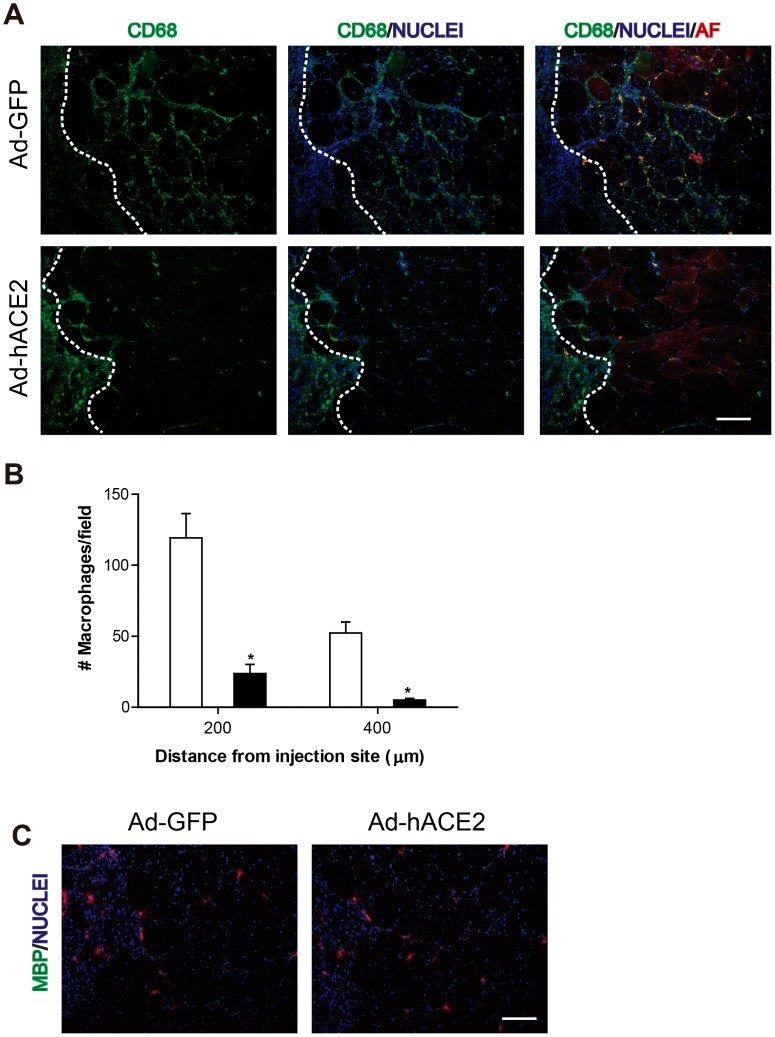
ACE2 overexpression reduces macrophage infiltration in dystrophic muscles. (A) To evaluate the direct effect of ACE2 *in vivo*, TA muscles from *mdx* mice were injected with Ad-GFP or Ad-hACE2 and analyzed after 5 days by immunofluorescence using anti-CD68 antibody (green) to determine macrophage infiltration 200 μm from the injection site (delimitated area). Nuclei were stained with Hoechst and as a reference point the muscles were overexposed to visualize the muscle fibers (red). (B) Quantification of macrophage infiltration into the muscle at 200 and 400 μm from the injection site. Infiltration in Ad-GFP injected muscle (white bars) and Ad-hACE2 (black bars) was compared. (C) Immunofluorescence staining with anti-major basic protein antibody to determine the number of eosinophils present in the muscle after injection. Nuclei were stained with Hoechst. Bar 100 μm.

Inflammation is a hallmark of dystrophic muscle and is associated with fibrotic accumulation [9], [10]. It has been shown that ACE2 deficiency induces inflammation [9], [42]. Since the expression of hACE2 was highest at day 5 and we also observed lower levels of collagen I and fibronectin at this time point, we evaluated the effect of the overexpression of ACE2 on inflammatory cells by examining the number of cells positive for CD68 (a specific marker for M1 macrophages) in the muscle sections. Figure 6A (upper panel) shows a great number of macrophages in the TA after Ad-GFP injection. However, the number of macrophages present near the injection site (delimitated area) was obviously lower in TA injected with Ad-hACE2. Quantitative analysis of the number of macrophages versus the distance from the injection site showed that the number of M1 macrophages near or far from the Ad-hACE2 injection site was lower than in the Ad-GFP injected muscle (Fig. 6B). As a control, Figure 6C (lower panels) shows that the adenoviral injections did not affect the number of eosinophils present 5 days after injection. These results suggest that ACE2 might improve the dystrophic muscle phenotype by decreasing the number of inflammatory cells that infiltrate the muscle.

**Figure 7:**
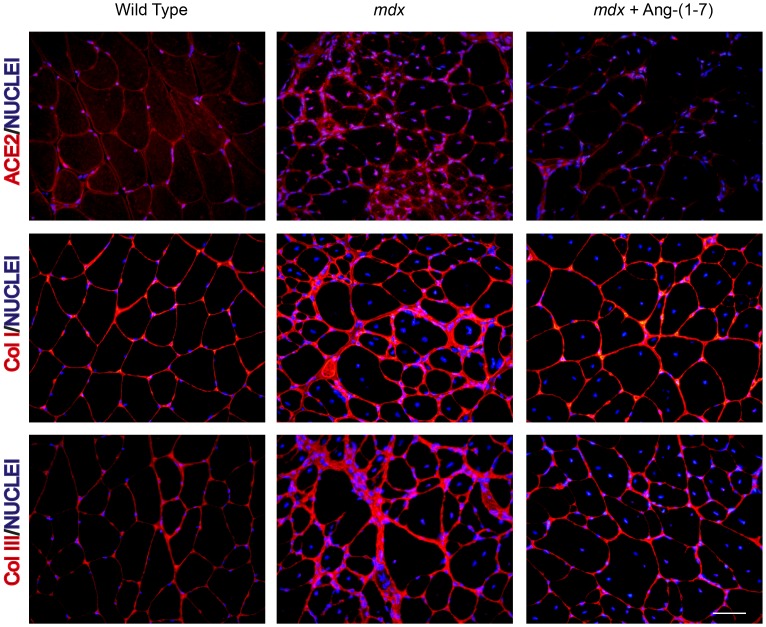
Angiotensin-(1-7) infusion reduces fibrosis and decreases ACE2 expression in *mdx* muscle. *mdx* mice were treated with Ang-(1-7) by systemic infusion for 8 weeks. ACE2, Col I, and Col III immunostaining in sections of GAST muscle from wild type (WT), *mdx,* and *mdx* mice treated with Ang-(1-7) is shown. Nuclei were stained with Hoechst. Bar 50 um.

We have detected a clear correlation among ACE2 levels and degree of fibrosis. Since Ang-(1-7) infusion reduces fibrosis [13] we asked whether the oppositive is true, we evaluated whether the levels of ACE2 are modulated as a result of peptide infusion. Immunolocalization studies (Fig. 7 upper panels) showed that the amount of ACE2 in *mdx* GAST was significantly lower in muscle infused with Ang-(1-7) compared to non-infused *mdx* tissue. This decrease in ACE2 staining was accompanied by a decreased number of interstitial nuclei. Figure 7 shows the concomitant decrease in collagen I and III (middle and lower panels, respectively). These results support the idea that ACE2 might function as a sensor of an inflammatory/fibrotic environment in the muscle tissue, with its expression being elevated in fibrotic situations or decreased when anti-inflammatory molecules are released.

Together, the results presented here suggest that muscle tissue maintains homeostasis by modulating ACE2 levels, such that ACE2 expression is increased in a pro-fibrotic environment or decreased in an anti-fibrotic one. Moreover, we provide novel evidence that augmenting ACE2 levels beyond the already elevated levels detected in the *mdx* muscle improves some aspects of the dystrophic phenotype, such as ECM deposition and infiltration of inflammatory cells.

## Discussion

The relevance of the RAS goes beyond its role in the physiology of the kidney and control of vascular tone. Considerable data suggest its importance in normal skeletal muscle function and tissue architecture [36], [43]–[45]. However, little is known about the regulation of the RAS components in skeletal muscle in the context of disease.

Ang-(1-7) acting through its Mas receptor plays a critical role in controlling skeletal muscle fibrosis in DMD [13]. ACE2, a key component of the alternative RAS, catalyzes the conversion of Ang II to Ang-(1-7); therefore, we investigated whether ACE2 expression and activity are subject to regulation in muscular dystrophy. ACE2 is normally expressed in skeletal muscle [46], [47]. Here, we report that ACE2 protein levels and concomitant activity are increased in dystrophic muscles compared with wt. It has been reported that the phenotype of *mdx* mice is milder than that of DMD [48]–[50]. It would be interesting to evaluate the level of ACE2 activity and expression of the Mas receptor in samples from DMD patients, since it could be hypothesized that in DMD ACE2 levels are not increased, given a more aggressive phenotype.

As a consequence of augmented ACE2 expression in the dystrophic muscle, we might expect increased local production of Ang-(1-7). Since Ang-(1-7) is an anti-fibrotic molecule, it might seem counterintuitive to have a simultaneous increase in ACE2 activity and excessive deposition of fibrotic constituents. However, results obtained from the analysis of *mdx*-Mas-KO muscle give some insight to help us understand these observations. Muscle from the double knockout mice exhibits a worse phenotype than *mdx*-derived muscle, with stronger TGF-β signaling, more fibrosis, and a further decrease in muscle function [13]. These findings suggest that production of endogenous Ang-(1-7) and its signaling through the Mas receptor are required to compensate for the progression of fibrosis in the *mdx* skeletal muscle. Thus, augmented ACE2 activity in dystrophic muscle or in wt muscle subjected to chronic damage seems necessary to protect the tissue from more severe damage such as that observed in the double knockout mouse. It will be helpful to quantify the amount of Ang-(1-7) generated in the skeletal muscle in future studies.

Following this line of reasoning, we hypothesized that we could reduce muscle fibrosis and inflammation by overexpressing ACE2 in dystrophic muscle. Indeed, we demonstrate for the first time that ACE2 gain of function correlates with decreased levels of fibrotic proteins and an important decrease in the number of inflammatory M1 macrophages, validating the anti-fibrotic and anti-inflammatory roles of ACE2 in skeletal muscle and supporting the idea of a beneficial effect of Ang-(1-7) in the context of disease. We expect that such a decrease in ECM content would improve muscle function because the decreased fibrosis observed in the *mdx* animals treated with Ang-(1-7) was associated with increased skeletal muscle force and better performance in endurance tests [13]. However, the adenovirus approach involving injection into the TA does not allow us to enrich all muscle fibers, therefore when we assayed muscle strength in isolated muscles injected with either Ad-GFP or Ad-hACE2 we did not observe significant differences (data not shown).

The membrane localization of ACE2 is relevant for its function. Here, we show that endogenous and overexpressed ACE2 protein was localized at the sarcolemma of individual fibers. It has been reported that ACE2 located at the plasma membrane enhances cell adhesion in an integrin-dependent manner. Indeed, soluble ACE2 (sACE2) is capable of suppressing integrin signaling mediated by FAK. It has also been shown that integrins are involved in the regulation of fibrosis, suggesting that pharmacologic targeting of all αv integrins may have clinical utility in the treatment of patients with a broad range of fibrotic diseases [51]. It would be interesting to determine whether sarcolemmal ACE2 participates in muscle fiber adhesion.

Under fibrotic conditions (*mdx* mouse and fibrosis induced in wt mice by chronic damage), ACE2 is present in the interstitial space and most likely associated with the plasma membrane of ECM-producing cells, myofibroblasts, and/or inflammatory cells [13]. As mentioned above, ACE2 exists in a soluble form in the plasma when shed from the cell membrane. Studies have linked elevated levels of sACE2 to myocardial dysfunction in heart failure patients [52], [53]. Moreover, ADAM17-mediated shedding of membrane ACE2 contributes to the development of neurogenic hypertension [54]. The authors of these studies proposed a protective role for ACE2 when it is associated with the plasma membrane, which is consistent with the findings presented here. The large amounts of ACE2 in the interstitial space lead us to speculate about a signaling role of ACE2 in addition to its catalytic role. In this vein it has been demonstrated that overexpression of ADAM17 has a deleterious effect in *mdx* mice [55] and mice with laminin alpha2- deficient muscular dystrophy [56].

It is intriguing that ACE2 activity is higher in skeletal muscles that exhibit more fibrosis in both the *mdx* mouse and the model of chronic induction of fibrosis. One plausible explanation is that some of the enhanced total ACE2 activity corresponds to enzyme associated with ECM-producing cells. This could be experimentally verified in dystrophic models in which fibrosis can be reduced [13], [37]. In fact, our results in *mdx* mice infused with Ang-(1-7) support this idea because ACE2 staining was lower than in non-treated *mdx* mice. Another explanation is that ACE2 activity is enhanced as a compensatory mechanism to produce more Ang-(1-7) and therefore decrease the amount of fibrotic proteins.

Fibrosis is a deleterious feature of several chronic diseases including DMD. Understanding the cellular and molecular mechanisms underlying muscle fibrosis is essential to develop effective antifibrotic therapies for DMD. Administration of Ang-(1-7) via systemic infusion has been shown to be a promissory approach for muscular disorders [13]. However, given its short half-life in plasma and its putative effects in other organs, we believe that modulating ACE2 activity in the skeletal muscle to increase local skeletal muscle levels of Ang-(1-7) may represent a new therapeutic approach.

In conclusion, this work shows for the first time that ACE2 protein levels and activity are augmented in fibrotic models of skeletal muscle and that a further increase in ACE2 activity reduces muscle fibrosis and infiltration of inflammatory cells *in vivo*.

## Supporting Information

Figure S1 **Functional expression of human ACE2 in HEK293T cells.** (A) Kinetics of substrate cleavage by cell extracts of non-infected HEK293T cells (white squares), and cells infected with Ad-GFP (gray squares) or Ad-hACE2 (black squares). (B) Hydrolysis rate of 50 uM Mca-YVADAPK(Dnp) by HEK293T cell extracts. (C) Detection of ACE2, GFP, and tubulin in HEK293T cell extracts by western blotting. Each lane was loaded with 20 μg of cell extract from non-transduced (NT) cells or cells transduced with Ad-GFP or Ad-hACE2. Molecular weight standards are shown on the right.
(TIF)

Figure S2 **ACE2 overexpression decreases fibronectin protein levels in dystrophic muscle.** (A) Effect of augmented expression of ACE2 on fibrosis was determined by western blot analysis. TA *mdx* extracts were obtained 1, 3, and 5 days after injection with PBS, Ad-GFP, or Ad-hACE2. Each lane was loaded with 50 μg of muscle extract and immunodetection of ACE2, fibronectin (FN), and GAPDH (loading control) was performed. Molecular weight standards are shown on the right. (B) TA *mdx* muscle was injected with Ad-GFP or Ad-hACE2 adenoviral vectors and analyzed after 1 or 5 days by ACE2 immunodetection. ACE2 expression was evident 5 days post-injection (Bar 200 um).
(TIF)
