# Supplementary figures and images for: ACE2 Is Augmented in Dystrophic Skeletal Muscle and Plays a Role in Decreasing Associated Fibrosis
Source: PLoS One. 2014 Apr 2;9(4):e93449. doi: 10.1371/journal.pone.0093449 (PMC3973684; doi:10.1371/journal.pone.0093449)

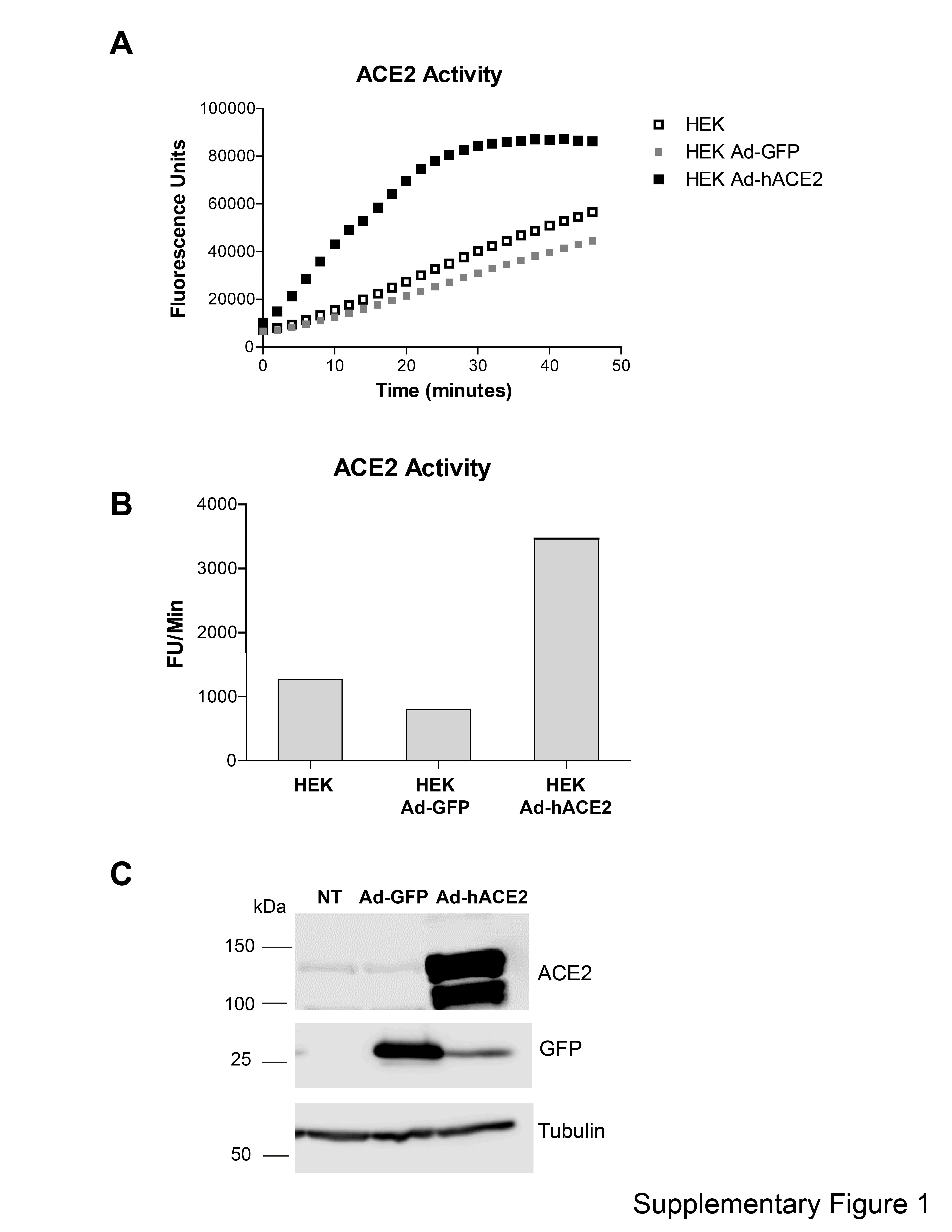

Supplement: Figure S1 — Functional expression of human ACE2 in HEK293T cells. (A) Kinetics of substrate cleavage by cell extracts of non-infected HEK293T cells (white squares), and cells infected with Ad-GFP (gray squares) or Ad-hACE2 (black squares). (B) Hydrolysis rate of 50 uM Mca-YVADAPK(Dnp) by HEK293T cell extracts. (C) Detection of ACE2, GFP, and tubulin in HEK293T cell extracts by western blotting. Each lane was loaded with 20 μg of cell extract from non-transduced (NT) cells or cells transduced with Ad-GFP or Ad-hACE2. Molecular weight standards are shown on the right. (TIF) [file pone.0093449.s001.tif]

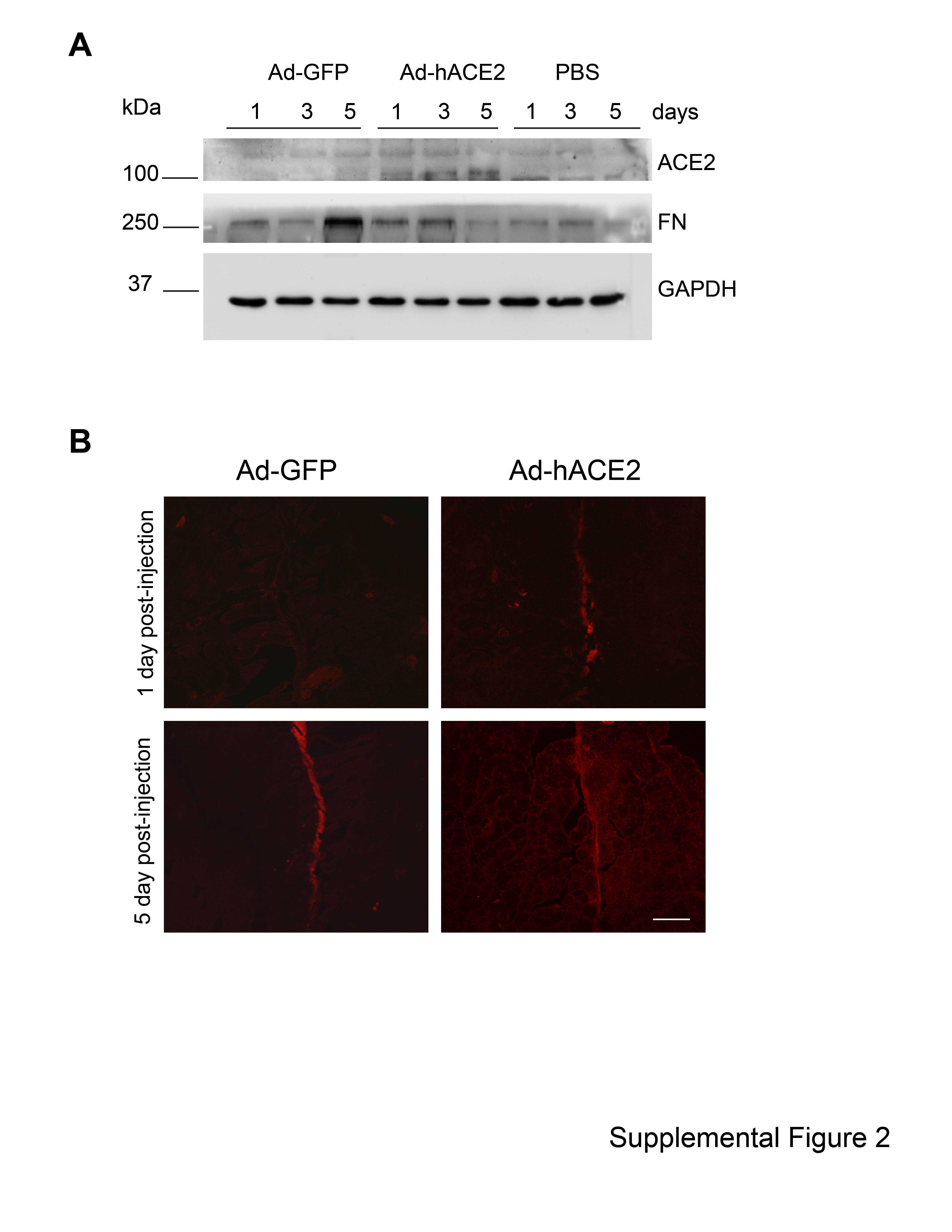

Supplement: Figure S2 — ACE2 overexpression decreases fibronectin protein levels in dystrophic muscle. (A) Effect of augmented expression of ACE2 on fibrosis was determined by western blot analysis. TA mdx extracts were obtained 1, 3, and 5 days after injection with PBS, Ad-GFP, or Ad-hACE2. Each lane was loaded with 50 μg of muscle extract and immunodetection of ACE2, fibronectin (FN), and GAPDH (loading control) was performed. Molecular weight standards are shown on the right. (B) TA mdx muscle was injected with Ad-GFP or Ad-hACE2 adenoviral vectors and analyzed after 1 or 5 days by ACE2 immunodetection. ACE2 expression was evident 5 days post-injection (Bar 200 um). (TIF) [file pone.0093449.s002.tif]
